# A Word to Our Exchanges

**Published:** 1853-01

**Authors:** 


### EDITORIAL DEPARTMENT.

***A word to our Exchanges.***—In this, the first number of a new year, we pause a moment, to bid God speed to our contemporaries, and to wish them success in their efforts to extend the domain of knowledge. As a general thing, we are happy to say that we can speak most favorably of them, and to all, and to each, we most heartily wish a happy, a successful, and a useful New Year.

*The American Journal of Medical Sciences,* comes to us, as usual, full of interest and instruction. The quarterly summary is unusually rich and varied, and demands the careful attention of the reader. The Review of the Transactions of the American Medical Association, is an excellent synopsis of the contents of that valuable publication.

Among the original communications, under the head of memoirs and cases, we notice a careful, and elaborate, investigation of the microscopical characters of cancer, in their application to diagnosis, by our fellow townsman, Dr. F. Donaldson. Dr. D. goes into a defence of the microscope, a course, which we regret to acknowledge, is still necessary, in spite of the great benefits which have accrued to physiology, and pathology, from its use. He then proceeds to the analysis of the various forms which he regards as characteristic of the disease, and illustrates these by numerous sketches from nature. He regards the difficulty in the recognition of the cancer-element, experienced by former observers, as due to the low magnifying powers which have heretofore been used by most microscopists who have investigated this subject. The article is eminently worthy an attentive perusal.

The account of the last illness, and the autopsy of Daniel Webster, is also extremely interesting. The unusual weight of the brain, would furnish an opportunity for a most pompous phrenological triumph, had not Mr. Amos Lawrence's brain, been most inopportunely discovered to have been two ounces heavier.

Our space will not allow us to make further allusions to the communications.

*The Medical News and Library,* is a cheap and useful journal, it contains much information in regard to medical matters, and publishes, continuously, some standard work on medicine. It has just finished Simon's Lectures on General Pathology, and has recommenced the publication of Todd and Bowman's admirable treatise on General Anatomy.

*The Medical Examiner and Record of Medical Science,* is another of our Philadelphia contemporaries which deserves a more extended notice than we are able to give it. During the past year, it has published some exceedingly valuable papers, among which we may mention Brown Sequard's Experimental Researches. The clinical reports, in this Journal, are freer than usual from the common place twaddle which disgraces so many of these publications.

*The Dental News Letter,* of the same city, contains varied and interesting information on the different subjects which most interest a dentist.

*The Semi-Annual Dental Expositor,* is edited by a poetical dentist, Solyman Brown, A. M., M. D., who treats us, in the November number, to a treatise on Dental Hygeia, in blank verse, and promises, for May, another on Mechanical Dentistry, in prose, with colored illustrations. The Expositor hails from No. 1 Spruce-st., New York.

*The New York Dental Recorder,* is a monthly publication, which contains a large number of valuable original communications on dental matters.

*The New York Journal of Medicine and the Collateral Sciences,* is a publication which merits the highest praise. The periods at which it is issued, (every other month, like Silliman's Journal,) recommend it to many who want time for the perusal of the larger quarterlies, and the more frequent monthlies. The publication of Orfila's admirable article on Nicotine, has, no doubt, been gladly received by American chemists.

*The New Jersey Medical Reporter, and Transactions of the New Jersey Medical Society,* is a spicy periodical. It is a decided enemy to quackery, and the January number contains two or three sharp articles on this subject, which ought to be read by medical men, and handed to their patients, especially to the parsons.

*Boston Medical and Surgical Journal.* This is always welcome to our table. It is ably edited, and has a large and intelligent corps of contributors. In our own city, it is one of the most popular of our medical periodicals.

*Stethoscope and Virginia Medical Gazette.* This Journal, published in Richmond, has just completed its second year. We admire the spirit of the Editor, and the nonchalant manner in which he dismisses those who complain of the way in which his periodical is conducted. The last number contains a really valuable article on the action and use of sulphate of quinine.

*Southern Medical and Surgical Journal.* Dr. Dugas, Professor of Surgery, in Augusta, Ga., edits this periodical. The December number contains an interesting report of the committee on Surgery, to the State Medical Society.

*The Charleston Medical Journal and Review,* is issued every two months. The selections are copious and judicious, and of the original articles, our attention has been particularly attracted, by the report of the Medical Committee on the endemic diseases of Newbery District, published in the November number.

*The New York Medical Gazette,* edited by Dr. Reese, comes to us in an altered, and, we think, an improved form. It has been changed to a monthly, and makes a very neat appearance, but it has not changed its character of an active, and uncompromising opponent of *quackery,*with, or without, a diploma.

*The North Western Medical and Surgical Journal,* hails from Chicago. Its editors appear to be industrious and able.

*The Western Medical and Chirurgical Journal,* comes from still nearer sundown. Keokuk, Iowa, is the place of its nativity, and the November number, is marked Vol. II, No. 8. Well done for Keokuk.

*The New Hampshire Journal of Medicine,* has the advantage of being conducted by an editor of no little ability. It is a good periodical.

*The Dental Register of the West,* is a small quarterly publication, issued at Cincinnati. Its pages are well filled with matters of interest to the dental profession.

*The Nashville Journal of Medicine and Surgery,* has been exciting no little interest among the profession during the past year, by the publication of the very entertaining and instructive European letters of Professor Paul F. Eve, a well known and highly esteemed surgeon of the south, and one of the editors of the periodical in which these letters appear. We have only received three numbers.

*The East Tennessee Record of Medicine and Surgery,* is published in Knoxville, Tennessee. edited by Dr. Frank A. Ramsey. We have only received one number, and have not had time to examine its contents. We welcome it, however, to the list of our exchanges.

*The Western Journal of Medicine and Surgery,* contains an elaborate article on Asiatic Cholera, as it appeared in New Albany, Indiana, in the year 1852, by Dr. Leszczynski, in which an ingenious parallel is drawn between this disease and malignant intermittent fever. We have often wondered that more stress was not laid upon the manifest resemblance of pathological conditions in the two diseases.

*The Ohio Medical and Surgical Journal,* published at Columbus, Ohio, is one of the neatest of our Western exchanges. It is well supplied with numerous original articles.

*The St. Louis Medical and Surgical Journal,* speaks well for the intelligence of medical news in Missouri. In the last number, the editor runs a tilt at the physiological absurdities of Mrs. Willard, backed, as they have been lately, by Dr. Cartwright and his crocodile, which latter animal bids fair to enjoy a reputation as extensive, and as apocryphal, as that of Mr. Waterton's alligator.

*The Buffalo Medical Journal,* is edited by Dr. Austin Flint, a true medical philosopher, whose articles are characterized by a cautious spirit of induction, and a candor worthy of the highest praise.

*The Canada Medical Journal,* is a good periodical.

*The American Journal of Insanity,* is too well known to need commendation.

*Nelson's Northern Lancet,* of Plattsburg, N. Y., contains much of interest to the medical profession, the only objection that can be urged against it, is what most readers would consider an undue prominence in its pages, given to the sayings, and doings, in the University of New York.

FOREIGN.

*The British and Foreign Medico-Chirurgical Review,* maintains its ancient reputation. The last number for 1852, is unusually attractive. The review of the report of the general board of health on quarantine and yellow fever, takes up the Boa Vista question again, and convicts the board of health of gross carelessness in the examination of the evidence, and extreme partiality in the review of it. The editors announce a new feature in the coming year, which cannot fail to meet the approbation of the profession. They propose to open its pages to original communications of merit. The value of this quarterly would be enhanced, if the American publishers were a little more prompt in its publication, and a little more regardful of neatness in its getting up. They, however, on their part, offer inducements, in the way of prizes of valuable medical books, to those who extend the circulation of the Review.

*Dublin Quarterly Journal of Medical Science.* This is one of the ablest of our Foreign exchanges, and a faithful chronicler of the progress of Irish medical practice, which has always been very highly esteemed in the United States. The number for August, (the last, by the way, which we have received,) contains Dr. Banon's article on hermaphrodism, which has been so largely copied into our medical journals, illustrated by two excellent lithographic plates. It reports one of the most remarkable cases on record.

*The London Lancet*, for November, has been received. It continues Coulson's Lectures on Lithotomy and Lithotrity, and Guthrie's on some of the more important points in surgery. The Clinical Lectures, by Hilton, Solly, and Adams, are interesting, and the general contributions, and selections, present the usual variety.

*The London Medical Gazette*, is another of our highly valued exchanges. The publication of lectures on various departments of medical science, of discussions in the different societies of the metropolis of the world, of numerous original papers, and of the latest medical intelligence, render it a very desirable visitor to the office of the practitioner, the library of the student, or the table of the editor.

*The Provincial Medical and Surgical Journal*, has, during the past year, been publishing a series of very full lectures on the diseases of children, by Dr. Merei. In it are to be found the transactions of the various Provincial societies.

*The Dublin Medical Press*, is an excellently conducted journal. It is always most welcome to our table. The last number, we have received, contains a most excellent article on malignant pustule, as it has appeared at Gibraltar.

